# Use of the Glycolipopeptid Biosurfactant Produced by *Lactiplantibacillus plantarum* Tw226 to Formulate Functional Cinnamon Bark Essential Oil Emulsions

**DOI:** 10.3390/foods14091540

**Published:** 2025-04-28

**Authors:** Virginia M. Lara, María F. Gliemmo, Marisol Vallejo, María del Carmen García González, María del Carmen Alfaro Rodríguez, Carmen A. Campos

**Affiliations:** 1 Departamento de Industrias, Facultad de Ciencias Exactas y Naturales, Universidad de Buenos Aires, Buenos Aires C1428EGA, Argentina; virginiamlara@gmail.com (V.M.L.); mfg@di.fcen.uba.ar (M.F.G.); 2 Instituto de Tecnología de Alimentos y Procesos Químicos (ITAPROQ), Facultad de Ciencias Exactas y Naturales, Ciudad Universitaria, Consejo Nacional de Investigaciones Científicas y Técnicas, Buenos Aires C1428EGA, Argentina; 3 Cátedra de Biología Celular y Molecular, Facultad de Ciencias Naturales y de la Salud, Universidad Nacional de la Patagonia San Juan Bosco, Comodoro Rivadavia U9005CXC, Argentina; soltrelew@gmail.com; 4 Departamento de Ingeniería Química, Calle Virgen de África, 7 (Escuela Politécnica Superior), 41011 Sevilla, Spain; mcgarcia@us.es (M.d.C.G.G.); alfaro@us.es (M.d.C.A.R.)

**Keywords:** biosurfactant, cinnamon bark essential oil, functional, lactic acid bacteria, glycolipopeptide, emulsions

## Abstract

The stabilization of essential oils in emulsions using surfactants of natural origin is of significant interest, and the use of biosurfactants produced by lactic acid bacteria could be an alternative. In this study, the total and partial substitution of Tween 80 in cinnamon bark essential oil emulsions was proposed using a glycolipopeptide biosurfactant produced by *Lactiplantibacillus plantarum* Tw226. The oil-in-water emulsions formulated contained cinnamon bark oil at a concentration of 5 g/L, with Tween 80, the biosurfactant, or a mixture of both as the surfactant agent, reaching a final concentration of 5 g/L. Homogenization was performed using a high-speed homogenizer. The emulsion with both the biosurfactant and Tween 80 was classified as a nanoemulsion (Z-av < 200 nm) that was stable for eight weeks, while the one with only the biosurfactant was a mini-emulsion (200 > Z-av < 500 nm). Furthermore, the emulsion with a combination of surfactants exhibited antioxidant activity equal to that of the emulsion with only Tween 80 and higher than that of the emulsion with only the biosurfactant. The antifungal activities of the three emulsions against *Candida tropicalis*, *Candida krusei*, and *Zygosaccharomyces bailii* did not change, regardless of the surfactant used, according to MIC values. In conclusion, a mixture of biosurfactant and Tween 80 or biosurfactant alone is an alternative for reducing or substituting synthetic surfactants in essential cinnamon bark oil emulsions, depending on their desired physical and functional properties. This work amplifies the scarce knowledge of essential oil emulsions stabilized with biosurfactants produced by lactic acid bacteria.

## 1. Introduction

Essential oils (EOs) are demanded by different markets, such as food and beverages (35%), fragrances, cosmetics, aromatherapy (29%), households (16%), and pharmaceuticals (15%) because of their fragrance, antioxidant, and antimicrobial properties [1]. They have been shown to effectively prevent microbial spoilage and oxidation in a diverse range of foods and beverages, including fruits, vegetables, meat, and dairy products [2,3,4]. In the cosmetic industry, they are useful because of their fragrance and preservation properties, which allow the elimination of synthetic components in formulas [5]. Additionally, most EOs are safe for consumers and have been granted a Generally Recognized as Safe (GRAS) status by the Food and Drug Administration (FDA). Among essential oils, cinnamon essential oil is rich in bioactive compounds such as cinnamaldehyde, eugenol, and linalool, which possess strong antimicrobial, antifungal, and insecticidal properties. Furthermore, it exhibits low toxicity, and it can be sensory-compatible with different food products [6]. The application of EOs in formulations is typically proposed as an oil phase in an emulsion because they are insoluble in water; therefore, it is necessary to stabilize EOs with surfactants [7]. The surfactants most commonly used as EOs emulsifiers have synthetic origins, such as those of the polysorbate or sorbitan ester family [8]. However, formulations that combine EOs with synthetic surfactants have the disadvantage of not being 100% natural, contrary to the current trend [9]. To advance the investigation of functional emulsions with natural ingredients, it is necessary to search for natural surfactants such as EO emulsifiers, excluding synthetic ones.

Biosurfactants are surfactants of natural origin that are non-toxic, biodegradable, and possess emulsifying, antimicrobial, and antibiofilm activities. They can also be produced from agricultural waste [10]. In the search for alternative food additives, biosurfactants from lactic acid bacteria (LAB) are of particular interest because they are GRAS, and their application in formulations is more feasible [11]. *Lactiplantibacillus plantarum* Tw226, a LAB isolated from *Odontesthes platensis*, has been identified as a cell-bound biosurfactant producer, being able to decrease the surface tension to 42.5 mN/m. This biosurfactant has a glycolipopeptide nature and is useful as an emulsifier for emulsions containing food ingredients such as corn oil, NaCl, and glucose [12,13]. Furthermore, the biosurfactant in an aqueous solution behaved as a Newtonian fluid exhibiting low viscosity, ruling out its action as a stabilizer by increasing the viscosity of the aqueous phase and concluding that it acts as an emulsifier [14].

Studies on biosurfactants as emulsifiers of EOs emulsions are scarce and are even less focused on biosurfactants produced by LAB [15]. Furthermore, the bioactivity of EOs can be modified by interactions between different ingredients [16]. For these reasons, it is necessary to study the stability and functional properties of emulsions formed with biosurfactants and to find natural surfactants that do not alter EOs properties. Few studies on EOs emulsions emulsified with biosurfactants produced by LAB are promising. For instance, a biosurfactant produced by *Lactiplantibacillus pentosus* was found to generate a more stable emulsion than polysorbate 20 when used as an emulsifier for rosemary oil emulsions [17]. Similarly, a biosurfactant produced by *Lactobacillus paracasei* demonstrated good emulsification activity in emulsions of rosemary oil, almond oil, jojoba oil, and wheat germ oil [18]. Therefore, the aim of this study was to evaluate the ability of biosurfactant produced by *L. plantarum* Tw226 to emulsify cinnamon bark EOs and to evaluate its potential use as an antioxidant and antimicrobial additive, and consequently, to expand the knowledge about the use of biosurfactants as emulsifiers of EOs emulsions.

## 2. Materials and Methods

### 2.1. Chemicals and Culture Media

Essential oil (EO) of cinnamon bark, *Cinnamomum zeylanicum,* was from Euma (Buenos Aires, Argentina), Tween 80, 6-Hydroxy-2,5,7,8-tetramethylchromane-2-carboxylic acid (Trolox), and 2,2-diphenyl-1-picryl-hydrazyl (DPPH) were obtained from Sigma Aldrich (St. Louis, MO, USA). Glycerol was obtained from Biopack (Buenos Aires, Argentina). All culture media were purchased from Biokar (Biokar Diagnostics, Beauvais, France).

### 2.2. Microorganisms and Inocula Preparation

The biosurfactant producer, *Lactiplantibacillus plantarum* Tw226, was previously isolated from the intestinal tract of Argentinian silverside (*Odontesthes bonariensis*) and identified [12,13]. The indicator yeasts, *Zygosaccharomyces bailii* NRRL 7256, *Candida tropicalis*, and *C. krusei*, were isolated from the equipment used in the apple juice processing industry in Argentina [19]. All microorganisms were cryopreserved at −80 °C. For *L. plantarum* Tw226, the broth for cryopreservation was De Man Rogose and Shape (MRS), and for the yeasts, it was Sabouraud, both supplemented with 20% (*v*/*v*) glycerol. To produce the inocula of *L. plantarum* Tw226 and yeast, cryopreserved microorganisms were grown twice in MRS broth for 24 h at 37 °C or in Sabouraud broth for 24 h at 25 °C, respectively.

### 2.3. Biosurfactant Production

Biosurfactant production was performed according to the methodology proposed by Gudiña et al. [20]. The bacterial suspension was inoculated in 600 mL of MRS broth in a 1:100 proportion and incubated for 48 h at 37 °C in an orbital shaker at 120 rpm (Vicking, Buenos Aires, Argentina). Then, cells were centrifuged and washed twice with demineralized water. The washed pellet was resuspended in 100 mL of phosphate-buffer saline (PBS: 10 mM Na_2_HPO_4_, 1.8 mM KH_2_PO_4_, 2.7 mM KCl, and 150 mM NaCl with pH adjusted to 7.0) and incubated under agitation at 150 rpm for 2 h at 25 °C to release cell-bound biosurfactant. Subsequently, the cells were centrifuged (Eppendorf, Hamburg, Germany) to obtain the cell-free supernatant (SLC) that contains the biosurfactant, which was filtered using a sterile filter with a pore size of 0.22 µm. Finally, the filtered SLC was partially purified by dialysis, using a membrane with a pore size of 6–8 kDa (SpectraPor, Waltham, MA, USA) against MilliQ water for 24 h at 4 ± 1 °C. The dialyzed SLCs were frozen and lyophilized for 48 h using a lyophilizer (Rificor, Buenos Aires, Argentine).

### 2.4. Emulsion Formulations

Three o/w emulsions were formulated using, as an aqueous phase, (i) a solution of Tween 80 at a concentration of 5.00 g/L (T), (ii) a solution of biosurfactant at a concentration 5.00 g/L (BS), and (iii) a solution with a mix of Tween 80 and biosurfactant at a concentration of 2.50 g/L each (BS:T). Cinnamon bark EO, at a concentration of 5.00 g/L, was used as the oil phase. An Ultraturrax T-25 high-speed homogenizer (IKA-Labortechnik, Staufen, Germany) was used to homogenize the systems. The homogenization conditions were 20 s at 13,500 rpm to favor mixing between the separate phases, followed by 50 s at 20,500 rpm to generate the formation of smaller droplets.

### 2.5. Characterization of the Emulsions

#### 2.5.1. Drop Size Analysis

The droplet size of the emulsions was determined by dynamic light scattering (DLS) using a Nano Zetasizer laser diffractometer (Malvern Instruments Ltd., Worcestershire, UK). The emulsions were stored at 25 °C and determinations were made every 14 days for a period of eight weeks. Droplet sizes were reported as the average volume (Z-average). Five readings were taken per sample, and each measurement was repeated on at least two independently prepared samples.

#### 2.5.2. Physical Stability

The physical stability of the emulsions was determined using a Turbiscan^®^ (Formulaction, Toulouse, France) using the multiple light scattering technique and evaluating the changes in the backscattering profiles (△BS%) as a function of time along 18 days of storage at 25 °C.

#### 2.5.3. Antioxidant Activity Determination

The antioxidant activity of the systems was determined using the DPPH (1,1-diphenyl-2-picrylhydrazyl) technique, according to Brand-Williams et al. [21] with slight modifications. An aliquot of 200 μL of the emulsions was added to 1.8 mL of DPPH 0.1 mM ethanolic solution. After incubation in the dark for 30 min at 30 °C, the absorbance was read at 517 nm (Shimadzu Corporation, Kyoto, Japan). As controls, solutions of biosurfactant and Tween 80 were used. The antioxidant activity was expressed as mmol Trolox/mL of emulsion.

#### 2.5.4. Antimicrobial Activity Determination

To evaluate the antimicrobial activity of the systems, their minimum inhibitory concentrations (MIC) against *Z. bailii*, *C. tropicalis*, and *C. krusei* in Sabouraud broth were determined by the dilution method using microplates. Briefly, serial dilutions of the emulsions were prepared starting from a concentration of 5000 ppm and using Sabouraud broth as a solvent. Subsequently, 180 µL of each dilution and 20 µL of the yeast inoculum with the concentration adjusted to 1.10^7^ CFU/mL were added to the wells, reaching a final concentration of 1.10^6^ CFU/mL. The concentrations of cinnamon oil in the wells were 562.5, 281.25, 140.4, and 70.2 ppm. The microplates were incubated for 48 h at 30 °C and the absorbance was registered at 600 nm (Abs600) at the beginning and the end of the incubation using an ELx808 microplate reader and processed using the Gen 5 software (BioTek Instruments, Winooski, VT, USA). The MIC was recorded as the minimum emulsion concentration that inhibits yeast growth (Abs600 value below 0.1) after 48 h of incubation.

### 2.6. Data Analysis

All assays were carried out in triplicate, and the data obtained from the different experiments were analyzed by means of one-factor Analysis of Variance (ANOVA), followed by Tukey’s test to detect significant differences at a significance level of 0.05. Results were presented as the mean ± standard deviation (SD). The analysis was performed using the statistical program Infostat (Infostat, 2020, University of Córdoba, Argentina).

## 3. Results and Discussion

### 3.1. Emulsions Characterization

#### 3.1.1. Droplet Size

**Figure 1:**
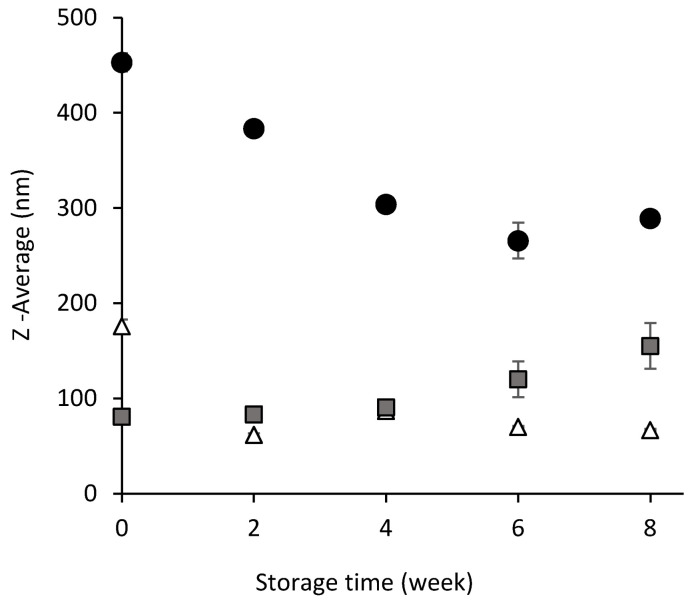
Evolution of the Z-average of the emulsions for 8 weeks stored at 25 °C. Emulsions stabilized with biosurfactant (●), a mixture of biosurfactant and Tween 80 in equal parts (△), and Tween 80 (■). In all cases, the final concentration of cinnamon bark essential oil and surfactant is 5000 ppm.

The average droplet size was measured to characterize and classify the emulsions. Figure 1 shows the changes in the Z-average of the emulsions during eight weeks of storage at 25 °C. Based on the Z-average distribution (Figure 1), T and BS:T emulsions can be classified as o/w nanoemulsions because their particle diameters are <200 nm [22]. Emulsions with smaller particle sizes, such as nanoemulsions, are generally preferred not only because of their better physical stability but also because smaller bioactive particles are easily absorbed [23]. Emulsion BS, in which only biosurfactant was used as an emulsifier, can be classified as a sub-micron or mini-emulsion, where the cut-off is extended to 500 nm [24].

The difference in the Z-average could be due to the differences between the chemical structures of biosurfactant and Tween 80. The surface tension of Tween 80 can reach a value of 0.004 mN/m [25], while the surface tension of the biosurfactant is 42.5 mN/m [13], which is a normal value for biosurfactant produced by LAB [10]. Zheng et al. [26] compared the formation of a Sea Buckthorn oil emulsion using isolates of soy protein, sodium casein, and sugar esters as emulsifiers. The emulsion formulated with soy protein, which has a globular and larger structure, presented the largest droplet diameter (465 nm) compared with the other two surfactants, which presented a smaller or more flexible structure. A similar phenomenon could occur in the studied case; because of its low molecular weight, Tween 80 can migrate quickly to the o/w interface and stabilize droplets; however, the biosurfactant is a glycolipidpeptide that presents a more complex structure, which probably hinders its diffusion to the interfaces. Consequently, the stabilizing interfacial layer, which prevents the favorable aggregation of the newly produced droplets, is formed later, leaving the droplets unprotected from aggregation [26].

Focusing on the drop size during storage, the combination of biosurfactant and Tween 80 in the BS:T emulsion resulted in a significantly lower droplet Z-average (*p* < 0.05) compared to the T emulsion, which utilized only Tween 80 as the emulsifying agent. Furthermore, the BS:T emulsion demonstrated stability in droplet size over time (*p* > 0.05), except during the initial formation phase. In the BS emulsion, the Z-average initially decreased and subsequently either remained stable for at least six weeks. Hashtjin and Abbasi [27] reported that a nanoemulsion made from orange EO and stored at 25 °C presented a decrease in Z-average until week 4 and then remained stable or increased, as in the samples used in our study. The authors indicated that this phenomenon could occur because of a gradual decrease in the barrier exerted by kinetic energy until a state of kinetic equilibrium was reached. In systems that include oil, water, and surfactants at room temperature, there is a kinetic energy barrier that prevents these systems from reaching a higher kinetic equilibrium state [22]. Reducing this barrier over time is likely to improve the kinetic equilibrium of the system. A similar trend was observed for lemongrass and cinnamon bark essential oil nanoemulsions emulsified with Tween 80 by Gonzalez et al. [28].

Unlike that observed in emulsions containing biosurfactants, the T emulsion exhibited an increase in Z-average with aging time. A basic problem with nanoemulsions containing EOs is Ostwald ripening because EOs components are highly soluble in water compared to medium- or long-chain triglycerides [29,30]. Ostwald ripening involves the diffusion of a dispersed phase from small to large droplets. This migration generates the disappearance of smaller droplets and the appearance of larger droplets [31], leading to an increase in the Z-average. This phenomenon depends not only on the solubility of the oil in the aqueous phase but also on the interfacial tension between the phases, according to the Lifshitz–Slyozov–Wagner theory, and both parameters vary with the concentration and nature of the emulsifier used [32,33].

#### 3.1.2. Turbidimetry Analysis

**Figure 2:**
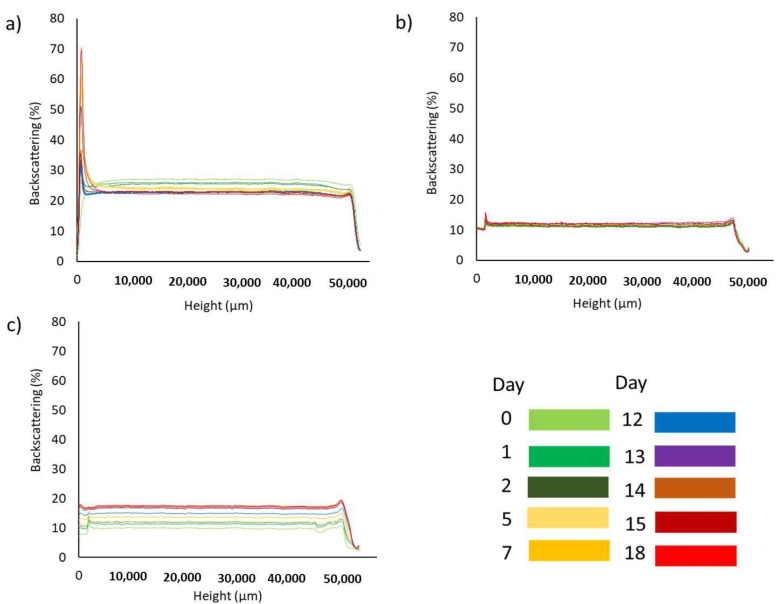
Evolution of the backscattering of emulsions stored at 25 °C for 18 days. Emulsions stabilized with biosurfactant (**a**), a mixture of biosurfactant and Tween 80 in equal parts (**b**), and Tween 80 (**c**). In all cases, the final concentration of cinnamon bark essential oil and surfactant is 5000 ppm.

Turbidimetry analysis over storage time is a useful methodology for studying the destabilization mechanisms of emulsions. Figure 2 shows the evolution of the backscattering light intensity of the emulsions (△BS%) versus the height of the sample and as a function of storage time at 25 °C.

First, it should be noted that all the samples were initially homogeneous, as can be deduced from the linear variation in backscattering at zero time. In addition, a low average backscattering intensity was observed as expected for slightly opaque products. In general, the sample containing biosurfactant exhibited an average backscattering intensity in the core of the emulsion of more than 27%, while that of the emulsion BS:T was 11%, and that of T was 10%. These differences may be attributed to the decrease in the average size of the droplets in the emulsion, as larger droplets scatter light more intensely than smaller droplets [34].

The turbidimetry results for the emulsion containing biosurfactant are shown in Figure 2a. The graph shows different instabilities. On the one hand, an increase in the △BS% with aging time can be observed at the bottom of the measuring cell, at a height of 0–2 mm, which is accompanied by a decrease at the top of the vial. This phenomenon can be attributed to the slight destabilization process by sedimentation. In principle, the appearance of sedimentation in an O/W emulsion is not usual; however, oil-in-water nanoemulsions may also be prone to sedimentation if the oil has a higher density than the water or if they contain small oil droplets covered by a relatively thick and dense shell [22]. The authors hypothesized that this mechanism is not significant because this sediment layer is small and hardly changes over time. However, a continuous decrease in the backscattering intensity in the middle of the measuring cell was observed. This result, according to Mengual et al. [35], indicates the existence of a variation in droplet size.

A similar behavior can also be observed for the BS:T emulsion (Figure 2b) emulsified with the biosurfactant-Tween 80 mix, although the changes observed at the bottom, middle, and top were clearly smaller. The △BS% profile did not show any noticeable changes with aging. This result was in agreement with the stability of the Z-average previously shown (Figure 1), suggesting that no destabilization processes occurred in the emulsion for 8 weeks at 25 °C. This is remarkable because the mixture of biosurfactant and Tween 80 allowed us to obtain a stable emulsion with a drop size similar to that of the emulsion containing only Tween 80, thus reducing the concentration of this synthetic surfactant by half.

Regarding the T emulsion (Figure 2c), only changes in the △BS% were observed over the storage time in the central part of the measuring cell, and these variations were significant. There was an increase in the △BS%, which, as in the BS emulsion, was associated with a variation in particle size. It should be highlighted that the increase in △BS% indicated that the droplet size was smaller than the incident light used with this technique.

**Figure 3:**
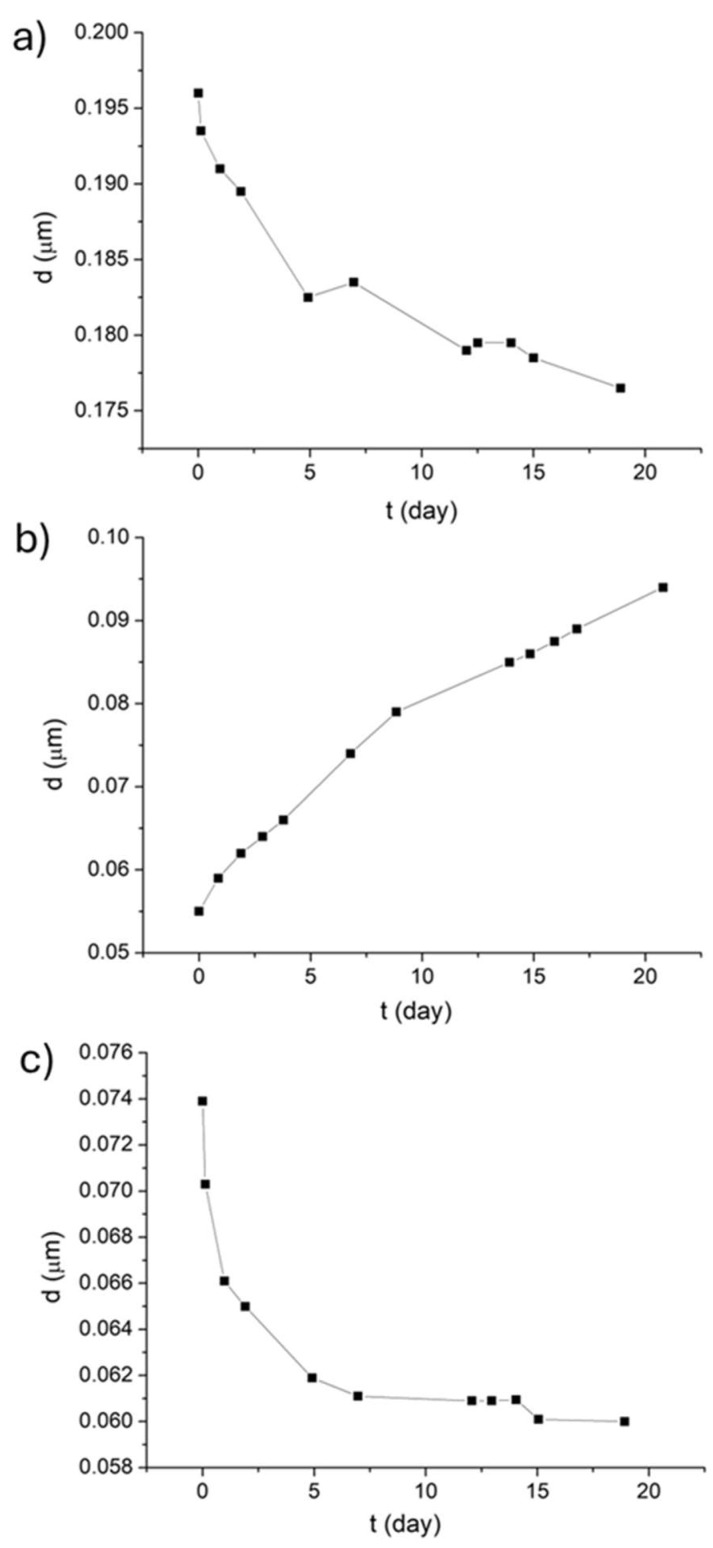
Evolution of the diameter (d) of emulsions for 18 days of storage (t) at 25 °C from results obtained by the multiple light scattering technique. Emulsions stabilized with biosurfactant (**a**), a mixture of biosurfactant and Tween 80 in equal parts (**b**). and Tween 80 (**c**). In all cases, the final concentration of cinnamon bark essential oil and surfactant is 5000 ppm.

As can be deduced from the results of multiple light scattering, the main destabilization mechanism of these emulsions is droplet size variation. The evolution of the droplet diameter with the aging time is also possible to analyze using this technique. As shown in Figure 3, the sample containing the biosurfactant showed a continuous decrease in diameter during the 18 days of the study (Figure 3a). In contrast, the emulsion containing Tween 80 showed a continuous increase (Figure 3b). The sample containing the mixture of biosurfactant and Tween 80 initially exhibited a decrease in size, but after 10 days of aging, the size remained stable (Figure 3c). The trends found were similar to those obtained by the dynamic light scattering technique, confirming the agreement between both techniques.

**Figure 4:**
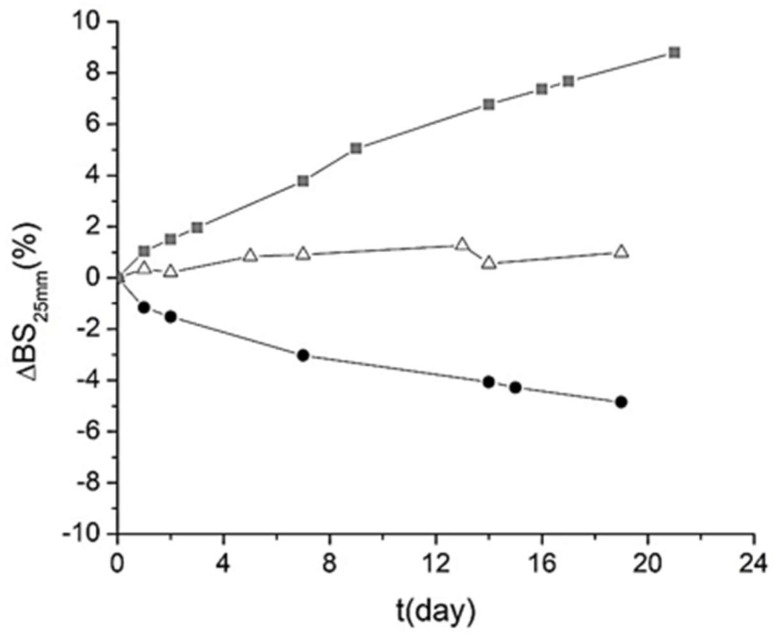
Evolution of the delta-backscattering at 25 mm of height (△BS_25mm_) for 18 days of storage (t) at 25 °C. Emulsions stabilized with biosurfactant (●), a mixture of biosurfactant and Tween 80 in equal parts (△), and Tween 80 (■). In all cases, the final concentration of cinnamon bark essential oil and surfactant is 5000 ppm.

To gain a deeper understanding of the physical stability, an analysis of the variation in △BS% value was carried out in the central zone of the measuring cell (25 mm sample height) as a function of aging time (Figure 4). Note that the △BS% is obtained by subtracting the backscattering at time 0 from the backscattering at time t. The emulsion exhibited the highest variation in △BS% (8.79 %), followed by the sample containing the biosurfactant (4.15 %) and the BS:T emulsion (0.99 %). Therefore, the sample containing the synthetic surfactant Tween 80 was the most unstable, whereas the mixture of Tween 80 and biosurfactant presented the lowest variation. Consequently, it can be concluded that BS:T was the most stable emulsion. Nevertheless, the emulsion containing the biosurfactant alone also showed acceptable stability, according to Muñoz et al. [36], who stated that a △BS% value below 5 % is indicative of stability in a sample.

According to the Z-average and the turbidimetry results, it could be considered that the biosurfactant works as an emulsifier and stabilizer of the emulsions. A similar behavior was previously reported for emulsions formulated with xanthan gum and corn oil, where biosurfactant did not play a role in the formation of smaller droplets, but it did play a role in their stabilization [13].

### 3.2. Functional Activity of Emulsion

#### 3.2.1. Antioxidant Activity

**Figure 5:**
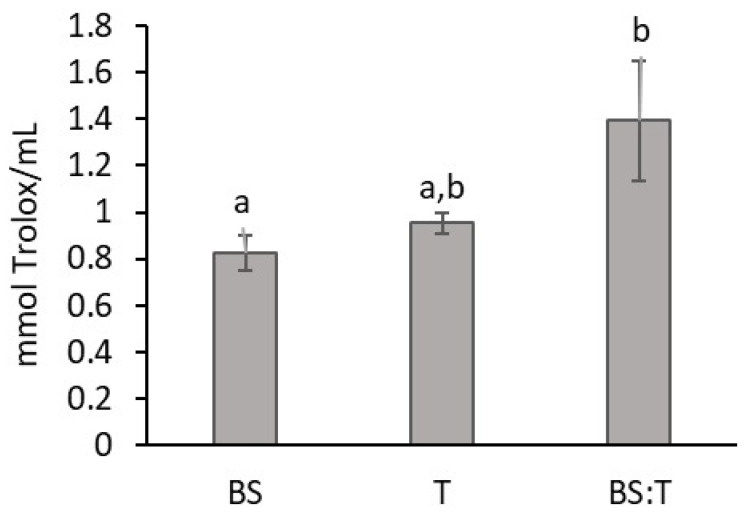
Antioxidant activity (mmol Trolox/mL) of BS, BS:T, and T emulsions. The averages whose superscripts have the same letter do not differ significantly (*p* < 0.05).

The antioxidant activities of the three emulsions and surfactant solutions are shown in Figure 5. The emulsions formulated with cinnamon bark oil had antioxidant activity, whereas the solution of surfactants (biosurfactant or Tween 80) did not; therefore, the antioxidant activity of the emulsions was attributed to the action of the EO. The antioxidant activity of the BS emulsion was compared with that of the BS:T and T emulsions. The BS emulsion did not show significant differences in mmol Trolox/mL with respect to the T emulsion, which presented a similar antioxidant activity. In contrast, the BS:T emulsion presented slightly higher antioxidant activity than the BS emulsion. The antioxidant activity of an agent in an o/w emulsion depends on its polarity and surface activity, and if the antioxidant agent is more dispersed at the oil-water interface, it will have a higher antioxidant activity [37]. In the case of an o/w emulsion, where the oily phase is an EO, antioxidant agents are present within the emulsion droplets. In this case, it could be argued that the system formulated in the emulsion BS:T presents a mechanism that favors the interaction between the antioxidant agent and molecules that tend to oxidize.

The emulsion containing only biosurfactant as an emulsifier allowed the elimination of Tween 80 in the formulation, and the emulsion BS:T reduced the concentration of Tween 80 and biosurfactant without impairing antioxidant activity and containing a lesser amount of the synthetic surfactant Tween 80.

#### 3.2.2. Antimicrobial Activity

The emulsions presented antifungal activity against the three evaluated yeasts, with no difference in MIC values, regardless of the surfactant used to formulate the emulsions. Solutions of Tween 80 and the glycolipopeptide biosurfactant did not exhibit antifungal activity; therefore, it must be considered that functionality is provided by the EO.

The MIC values of the emulsions against *Z. bailii* were 140.6 ppm. Despite the different emulsifying agents and variations in the droplet sizes of the emulsions, there was no change in the MIC value. Gonzalez et al. [28] reported a similar MIC for an emulsion of cinnamon bark EO, emulsified by Tween 80, against *Z. bailii,* and, in agreement with this work, they did not observe changes in the MIC value due to different droplet sizes. They also reported that Tween 80 did not inhibit the growth of *Z. bailii*.

The antifungal activity was also proved against *Candida tropicalis* and *Candida krusie*, strains that produce animal and human infections, found in several environments and recognized, in certain cases, as multidrug resistant [38,39]. The MIC against *C. tropicalis* was 563 ppm, and that against *C. krusei* was 281 ppm, independent of the surfactant used for stabilization. Dias de Castrol and Olivera Lima [40] reported that the MIC against *C. tropicalis* and *C. krusei* was 312.5 ppm. Even though the result for *C. tropicalis* is higher, it must be considered that the MIC value could be different depending on the strain of Candida used [41].

The antimicrobial activities of EOs are well known, and the action mechanisms are multiple. Especially for cinnamon bark essential oils, the mechanisms that work in this process are oxidative stress induction, ergosterol biosynthesis disruption, inhibition of ATPase activity, cell membrane disruption, and inhibition of hyphae formation [42]. Sometimes the presence of additives that emulsify the essential oils in formulations could affect the antimicrobial activity [43], but the results presented in this work show a promising panorama for the use of biosurfactants in antimicrobial formulations with antifungal activity, because they do not seem to interfere with antimicrobial mechanisms or interactions with yeasts, nor do they alter the MIC value.

## 4. Conclusions

The BS:T mixture was useful for formulating a nanoemulsion (Z-average <200 nm), which was stable for 6 weeks at 25 °C, with higher antioxidant activity. In contrast, the emulsion with only a biosurfactant as an emulsifier could be used to formulate a mini-emulsion (Z-average >200 nm, Z-average <500 nm) with low precipitation, which presents antioxidant activity equal to that of the emulsion with only Tween 80. Additionally, it must be stressed that the antifungal activity of emulsions against *C. tropicalis*, *C. krusei,* and *Z. bailii* was consistent regardless of the surfactant used.

In conclusion, the biosurfactant from *L. plantarum* Tw226 is useful for reducing the concentration of synthetic surfactants, such as Tween 80, or replacing it in functional EO emulsions, depending on the desired characteristics of the formulated emulsion, opening a door to the study of biosurfactants as emulsifiers in EO emulsions with possible application in several industries.

## Data Availability

The original contributions presented in the study are included in the article, further inquiries can be directed to the corresponding author.
